# Short time to blood culture positivity in *Enterococcus faecalis* infective endocarditis

**DOI:** 10.1007/s10096-021-04210-9

**Published:** 2021-03-09

**Authors:** Karl Oldberg, Rebecca Thorén, Bo Nilson, Patrik Gilje, Malin Inghammar, Magnus Rasmussen

**Affiliations:** 1 Section for Infection Medicine, Department of Sciences Lund, Lund University, Lund, Sweden; 2 Clinical Microbiology, Labmedicin, Region Skåne, Lund, Sweden; 3 Division of Medical Microbiology, Department of Laboratory Medicine, Lund University, Lund, Sweden; 4 Section for Cardiology, Department of Sciences Lund, Lund University, Lund, Sweden; 5 Skåne University Hospital, Lund, Sweden; 6 Division of Infection Medicine Diseases, Department of Clinical Sciences Lund, Lund University, BMC B14, SE-223 63 Lund, Sweden

**Keywords:** *Enterococcus faecalis*, Bacteremia, Time to positivity, Infective endocarditis

## Abstract

Time to blood culture positivity (TTP) is an indirect measure of bacterial concentration in blood. A short TTP has been linked to the presence of infective endocarditis (IE) and to poor prognosis in *Staphylococcus aureus* bacteremia. We analyze factors influencing TTP in bacteremia with *Enterococcus faecalis*. This retrospective observational study of medical records included adults diagnosed with monomicrobial *E*. *faecalis* bacteremia between 2015 and 2018 in the Skåne region (Sweden). For each episode, the shortest TTP was recorded. Median TTP was compared between patients grouped based on age, sex, comorbidity, site of acquisition, and focus of infection. Using a dichotomized TTP (shorter or longer than 12 h), a multivariable logistic regression for factors associated to TTP was performed. The association between TTP and IE or mortality was evaluated. Three hundred sixty-seven episodes with monomicrobial *E*. *faecalis* bacteremia with the corresponding TTP were identified. Median TTP for the entire cohort was 11.6 (IQR 9.9–14.1) h and a significantly shorter TTP was noted for episodes which represented IE (*n* = 55, 9.4 (IQR 6.4–10.6) h). Only IE remained associated with a short TTP (≤ 12 h) in binary logistic regression analysis. Factors associated with IE were investigated and TTP was associated with IE also when adjusted for age, gender, comorbidity, and nosocomial acquisition. There was no association between TTP and mortality. A low TTP is associated with IE in *E*. *faecalis* bacteremia and could be used as a help in determining the need for echocardiography in patients with this condition.

**Supplementary Information:**

The online version contains supplementary material available at 10.1007/s10096-021-04210-9.

## Introduction

Blood cultures are essential in the diagnostic work-up in patients with suspected bacterial infection. A positive blood culture will help both to achieve a correct diagnosis and to guide choice of treatment according to the antimicrobial susceptibility testing. Time to positivity (TTP), the time it takes for a blood culture to become positive, is readily available in automated blood culture systems. TTP is influenced by factors such as bacterial growth rate and volume of blood but can, for a given species, be regarded as an indirect measure of the concentration of bacteria in blood [1]. The clinical use of TTP is mainly the differential TTP between blood cultures drawn from central lines and peripheral blood, which can help to diagnose catheter-associated bloodstream infections [2]. It is only lately that data have gathered pointing to other potential uses of TTP in clinical decision-making [3].

A short TTP has been associated with poor outcome in bacteremia with *Staphylococcus aureus* [4–6], *Streptococcus pneumoniae* [7], *Pseudomonas aeruginosa* [8], and non-typhoid *Salmonella* [9]. Moreover, a short TTP is associated with an endovascular source of infection, including infective endocarditis (IE), in bacteremia with *S*. *aureus* [5, 10, 11]. Recently, we suggested that TTP is useful to determine the need for echocardiography in *S*. *aureus* bacteremia [12].

*Enterococcus faecalis* is part of the normal flora and a pathogen that can cause a range of clinical conditions including IE, urinary tract infections, intraabdominal infections, osteomyelitis, and soft tissue infections [13, 14]. *E*. *faecalis* bacteremia (EFsB) necessitates different diagnostic work-ups depending on the clinical condition suspected. If IE is suspected, the laborious procedure of transesophageal echocardiography (TEE) is warranted. In order to avoid unnecessary investigations, recent work has focused on identifying risk factors of IE in EFsB. These studies have collectively demonstrated a relatively high risk for IE in patients with monomicrobial EFsB [15–17] and have suggested unknown focus of primary infection and several positive blood cultures as risk factors for IE in enterococcal bacteremia [17–19]. Thirty-day mortality in EFsB is around 20% and higher in patients with an abdominal focus of infection and nosocomial infection [13].

This study was initiated to investigate which factors that are associated with the TTP in monomicrobial EFsB and whether TTP can predict the outcome in this clinical condition.

## Methods

### Settings and microbiology

This study was conducted in the Region of Skåne in southern Sweden, comprising 1.3 million persons. The region is served by a single microbiological laboratory that has satellite blood culture cabinets (BACTEC FX, Becton Dickinson, Franklin Lakes, USA) in the five largest hospitals, where blood cultures are put into the cabinets at all hours. Five smaller hospitals are without blood culture cabinets. Due to probable and unpredictable delays before vials from these hospitals were incubated, cases from hospitals without blood culture cabinets were not included. Cultures were incubated for a maximum of 120 h. Species identification in positive blood cultures was performed using Microflex MALDI-TOF MS (Bruker Daltronics, Bremen, Germany), with the software FlexControl and MBT Compass 4.1 and the reference database MBT Compass Library DB-7854.

### Episodes and definitions

All episodes of monomicrobial EFsB in patients ≥ 18 years old between 2015 and 2018 in the five hospitals holding culture cabinets were reviewed and clinical and microbiological records were collected retrospectively. Episodes of EFsB in the same region between 2012 and 2016 have previously been analyzed to generate the DENOVA score [19]. Episodes for which the medical records or TTP were not available were excluded from the study, as were episodes where antibiotic treatment had been administered within 7 days before the first blood cultures were drawn. For each episode of bacteremia, the shortest TTP of all the blood culture vials drawn within the first 24 h was recorded.

Parameters needed for the modified Duke criteria [20], the Charlson comorbidity index [21], and the previously described score to determine the risk for IE in EFsB (DENOVA) [19] were collected, as well as demographic variables and outcome measured as mortality in-hospital, at 30 and 90 days and recurrence of monomicrobial EFsB within 180 days. An episode of EFsB started with a positive blood culture and was concluded by either at least 7 days of effective antimicrobial treatment or after 30 days. IE was defined as definite IE according to the modified Duke criteria. Thus, only non-nosocomial acquired EFsB was regarded as a major criterion [20]. Echocardiography reports that were ambiguous were reevaluated blindly by an experienced echocardiographer (PG) and classified as either IE or non-IE. Other foci of infection were defined as fulfillment of at least two of the following criteria (a) typical signs or symptoms of infection, (b) isolation of *E*. *faecalis* at the site of infection, and (c) imaging results compatible with focal infection [19]. Cases not fulfilling at least two of these criteria, or definite IE, were considered to have an unknown focus of infection. A focal infection was also considered an origin of infection if it was the likely site of entry of the bacteria into the blood stream. Site of acquisition was classified as nosocomial if the positive blood culture was drawn after > 48 h of hospitalization, and otherwise as community acquired. Previous EFsB was defined as growth of *E*. *faecalis* in a blood culture within the previous 90 days. Patients were followed for a minimum of 180 days after the episode through the medical records which are common to the entire region.

### Statistical analyses

Statistical analyses were performed using SPSS Statistics version 26 (IBM) and Stata (StataCorp). TTP was dichotomized into TTP ≤ 12 or > 12 h which was close to the median and a practical cut-off. Patients were categorized into one of three age groups of similar sizes: 18–70, 71–80, and ≥ 81 years. Underlying comorbidity was categorized into three groups according to the Charlson score: 0–2, 3–4, and ≥ 5.

Median TTP and inter quartile range (IQR) was calculated according to grouping by age, gender, Charlson score, nosocomial vs. non-nosocomial acquisition, and focus of infection. The differences between groups were assessed with the Mann-Whitney or the Kruskal-Wallis test.

A cross-tabulation was made with dichotomized TTP as the outcome variable and variables that had a priori been postulated to possibly have an effect on TTP. Associations between the exposures and TTP were tested with the chi-square test. Logistic regression was used to estimate ORs for TTP ≤ 12 h, the following variables were included in the model: gender, age (grouped as 18–70, 71–80, and > 80 years of age), Charlson score (grouped as 0–2, 3–4, and ≥ 5), site of acquisition (community vs. nosocomially (positive blood culture drawn after more than 48 h of hospitalization) acquired), and site of infection (urinary tract, IE, gastro intestinal (GI) and biliary, skeletal or joint, other known focus of infection, or unknown focus of infection).

Another cross-tabulation was made with IE as the outcome, with TTP and other markers of risk as exposure variables. These variables were chosen based on prior knowledge [19]. Statistical significance tests using chi-square test was performed.

Multivariable logistic regression analysis was performed with IE as the outcome and TTP ≤ 12 h (yes/no) as the main predictor. These models were adjusted for age, gender, underlying comorbidity (Charlson’s comorbidity score), and site of acquisition. Both multivariable logistic regression analyses were first made with all episodes included and thereafter, as a sensitivity test, with only the first episode per patient included.

A ROC curve was plotted with IE as the outcome and TTP as the exposure.

TTP as a continuous exposure variable and the outcomes death in hospital, within 30 days, 90 days, and relapse of EFsB within 180 days, were cross tabulated and hypothesis testing was performed using the Mann-Whitney test.

## Results

### Description of the cohort

A total of 359 patients with EFsB fulfilling the inclusion criteria were identified. Of these, 23 were excluded due to antibiotics having been administered before the first blood cultures and 13 were excluded due to unobtainable records or TTP. This left 367 episodes in 323 patients in the analysis. Three patients had four episodes, six patients had three episodes, and 23 patients had two episodes each. Median time between end of antibiotic treatment and a new EFsB was 30 days (IQR 16–72). Median age was 74 years (IQR 69–83), 72% were male, median Charlson comorbidity score was 2 (IQR 1–4), and 84% of the episodes were community acquired (Table 1).

**Table 1:** Comparison of TTP between different groups

	TTP median, h (IQR)^a^	*p* value
Gender		
Female (104, 28%)^b^	11.6 (9.6–13.0)	0.34
Male (263, 72%)	11.6 (9.9–14.3)	
Age		
18–70 (112, 31%)	11.7 (9.7–14.5)	0.54
71–80 (135, 37%)	11.7 (10.0–13.8)	
81–96 (120, 33%)	11.2 (9.7–13.8)	
Charlson score		
0–2 (209, 60%)	11.6 (10.0–14.1)	0.15
3–4 (93, 25%)	11.5 (9.4–13.4)	
≥ 5 (65, 18%)	12.3 (10.2–15.6)	
Site of acquisition		
Community acquired (309, 84%)	11.5 (9.6–13.9)	0.043
Nosocomial (58, 16%)	12.3 (10.7–14.3)	
Focus of infection		
IE (55, 15%)	9.4 (6.4–10.6)	< 0.001
Urinary tract (136, 37%)	12.1 (10.6–14.4)	
GI and biliary (29, %)	12.4 (10.8–14.3)	
Skin and soft tissue (28, 8%)	12.1 (10.2–16.1)	
Skeletal and joint (15, 4%)	13.3 (11.5–20.7)	
Other known^c^ (10, 3%)	12.7 (9.7–15.4)	
Unknown (94, 26%)	11.7 (10.0–14.1)	

^a^Interquartile range

^b^Number of episodes and the share of episodes are given within parenthesis

^c^Pneumonia (*n* = 7) and other airway infections (*n* = 3)

Transthoracic echocardiography (TTE) and transesophageal echocardiography (TEE) were performed in 90 of the 367 episodes, while TTE alone was performed in 70 episodes.

### Time to positivity

Median TTP for the entire cohort was 11.6 (IQR 9.9–14.1) h. Median TTP together with IQR in different groups which could be related to TTP is given in Table 1. The TTP was not equal between the different types of infection with IE having the lowest median TTP (*p* < 0.001). A cut-off of 12 h was used to tentatively separate a short TTP from a long TTP. This cut-off was close to the median, and it could be easy to apply to clinical practice. The proportion of episodes with a short TTP was calculated for variables potentially associated with TTP and the results are given in Table 2. The only variable clearly associated with a short TTP in univariate analysis was IE, OR 12.8 (95% CI 4.4–37) (Table 2). IE remained associated with a short TTP in multivariate analysis, OR 13 (95% CI 4.4–38). As a sensitivity analysis, all recurrent episodes were excluded leaving only the first episode in each patient (*n* = 323). The results were similar, OR of 12 (95% CI 3.5–41) for TTP ≤ 12 h in IE. No other variables were associated with TTP ≤ 12 h (see supplementary table 1).

**Table 2:** Variables associated with a long or short TTP

Univariate analysis					Multivariable analysis^c^
	TTP ≤ 12h (*n* = 205)	TTP > 12h (*n* = 162)	*p* value^b^	OR univariate	OR (95% CI)
Gender					
Male (263, 72%)	142 (69)^a^	121 (75)	0.25	ref	ref
Female (104, 28%)	63 (31)	41 (25)		1.3 (0.82–2.1)	1.6 (0.96–2.7)
Age					
18–70 (112, 31%)	60 (30)	52 (32)	0.29	ref	ref
71–80 (135, 37%)	71 (35)	64 (40)		0.96 (0.58–1.6)	1.1 (0.62–1.9)
81–96 (120, 33%)	74 (36)	46 (28)		1.4 (0.83–2.4)	1.5 (0.84–2.7)
Charlson score					
0–2 (209, 57%)	118 (58)	91 (56)	0.084	ref	ref
3–4 (93, 25%)	58 (28)	35 (22)		1.3 (0.77–2.1)	1.4 (0.84–2.5)
≥ 5 (65, 18%)	29 (14)	36 (22)		0.62 (0.35–1.1)	0.75 (0.41–1.4)
Site of acquisition					
Community acquired (309, 84%)	177 (86)	132 (81)	0.21	ref	ref
Nosocomial (58, 16%)	28 (14)	30 (19)		0.70 (0.40–1.2)	1.0 (0.55–1.9)
Site of infection					
Urinary tract (136, 37%)	68 (33)	68 (42)	< 0.001	ref	ref
IE (55, 15%)	51 (25)	4 (2.5)		12.8 (4.4–37)	13.0 (4.4–38)
GI and biliary (29, 7.9%)	12 (5.9)	17 (11)		0.71 (0.31–1.6)	0.65 (0.27–1.5)
Skin and soft tissue (28, 7.6%)	14 (6.8)	14 (8.6)		1.0 (0.44–2.3)	0.96 (0.40–2.3)
Skeletal and joint (15, 4.1%)	5 (2.4)	10 (6.2)		0.50 (0.16–1.5)	0.96 (0.40–2.3)
Other known (10, 3.3%)^d^	4 (2.0)	6 (3.7)		0.67 (0.18–2.5)	0.57 (0.15–2.2)
Unknown (94, 26%)	51 (25)	43 (27)		1.2 (0.70–2.0)	1.1 (0.64–1.9)

^a^The number of episodes and the share of episodes are given within parenthesis

^b^Univariate test of significance was performed with the chi-square test

^c^The multivariable testing was performed using binary logistic regression. The following variables were included in the model: gender, age, Charlson score, site of acquisition, and site of infection

^d^Pneumonia (*n* = 7) and other airway infections (*n* = 3)

### Infective endocarditis

**Fig. 1:**
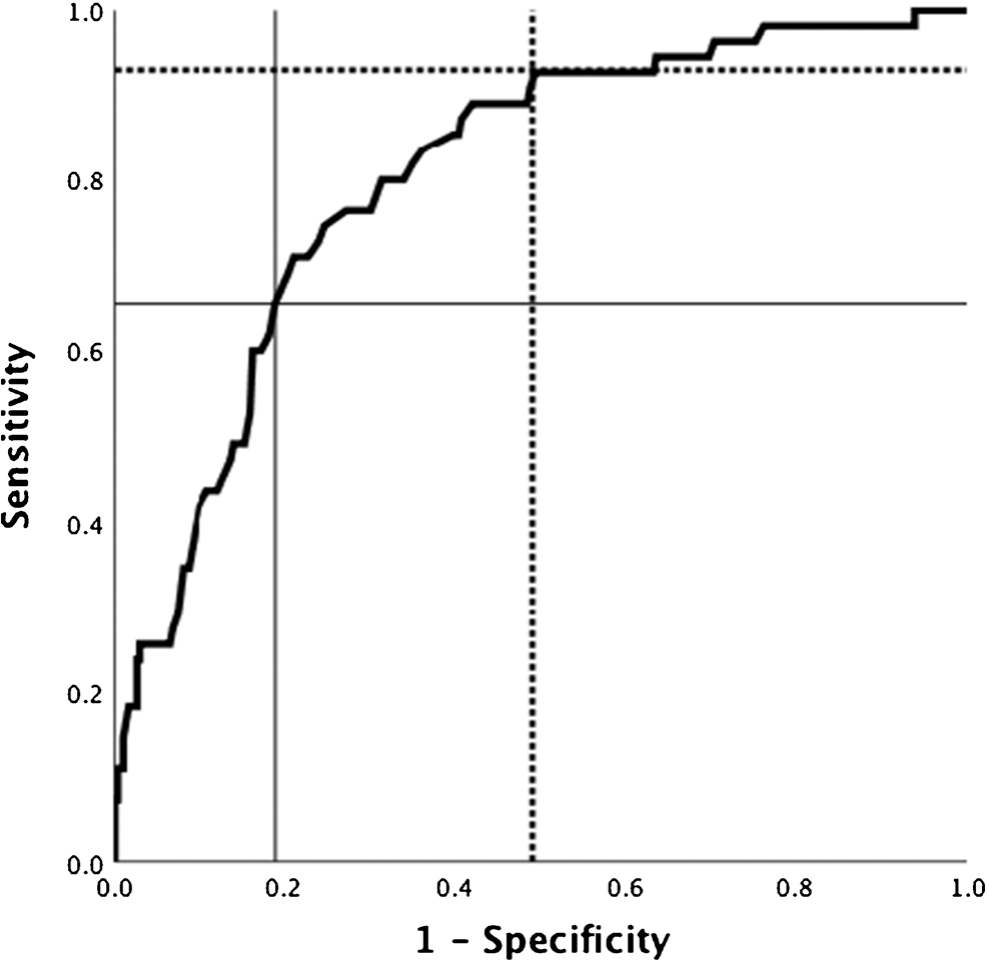
Receiver operator characteristics (ROC) curve for the ability of TTP to predict IE. The area under the curve (AUC) is 0.80 (95% CI 0.74–0.86). The dashed line shows the corresponding sensitivity and specificity at a cut-off of 12 h, whereas the filled lines show the consequence of a tentative cut-off at the lower IQR-limit (9.9 h)

To investigate how TTP was associated with IE, episodes fulfilling definite Duke criteria were compared with those episodes that did not. The receiver operator characteristics (ROC) curve for the ability of TTP to discriminate between episodes with and without IE is shown in Fig. 1. Area under the curve (AUC) was 0.80 (95% CI 0.74–0.86). With the cut-off used above at ≤ 12 h, the sensitivity for IE was 93% (CI 83–97) and the specificity 51% (CI 45–56). The positive likelihood ratio was 1.8 and the negative likelihood ratio was 0.15. If a cut-off at the lower quartile of the TTP distribution, ≤ 9.9 h, was chosen instead, the association to IE remained highly significant in univariate analysis using chi-square test. The sensitivity for IE decreased, while the specificity increased, see Fig. 1.

In Table 3 variables that are known or plausible markers of risk for IE are presented, with IE as the outcome. Univariate analyses within the cross table identified associations between IE and previous *E*. *faecalis* bacteremia, community acquisition, long duration of symptoms, embolizations, > 2 positive blood cultures, unknown origin of infection, heart valve disease, auscultation of murmur, and a short TTP. In multivariable logistic regression, the OR for IE in patients with TTP ≤ 12 h was 13.7 (95% CI 4.8–39.4) adjusted for age, gender, Charlson’s comorbidity score, and nosocomial acquisition.

**Table 3:** Comparison of episodes representing IE or non-IE

	IE (*n* = 55)	Non-IE (*n* = 312)	*p* value^b^	OR
Gender				
Male 263 (72)	43 (78)^a^	220 (71)	0.25	ref
Female 104 (28)	12 (22)	92 (29)		0.67 (0.34–1.3)
Age				
18–70 112 (31)	18 (33)	94 (30)	0.79	ref
71–80 135 (37)	18 (33)	117 (38)		0.80 (0.40–1.6)
81–96 120 (33)	19 (35)	101 (32)		0.99 (0.49–2.0)
Charlson score				
0–2 209 (57)	38 (69)	171 (55)	0.057	ref
3–4 93 (25)	13 (24)	80 (26)		0.73 (0.37–1.5)
≥ 5 65 (18)	4 (7.3)	61 (20)		0.30 (0.10–0.86)
Previous *E*. *faecalis* bacteremia				
No 318 (87)	37 (67)	281 (90)	< 0.001	ref
Yes 49 (13)	18 (33)	31 (9.9)		4.4 (2.2–8.7)
Site of acquisition				
Community 309 (84)	53 (96)	256 (82)	0.005	ref
Nosocomial 58 (16)	2 (3.6)	56 (18)		0.17 (0.04–0.73)
Duration of symptoms ≥ 7 days				
No 309 (84)	10 (18)	299 (96)	< 0.001	ref
Yes 58 (16)	45 (82)	13 (4.2)		104 (43–250)
Embolisation				
No 352 (96)	40 (73)	312 (100)	< 0.001	NA^c^
Yes 15 (4.1)	15 (27)	0 (0)		NA
Number of positive blood cultures ≥ 2				
No 126 (34)	4 (7.3)	122 (39)	< 0.001	ref
Yes 241 (66)	51 (93)	190 (61)		8.2 (2.9–23)
Origin of infection				
Known 189 (52)	1 (1.8)	188 (60)	< 0.001	ref
Unknown 178 (48)	54 (98)	124 (40)		82 (11–600)
Valve disease				
No 280 (76)	17 (31)	263 (84)	< 0.001	ref
Yes 87 (24)	38 (69)	49 (16)		12 (6.3–23)
Auscultation of murmur				
No 297 (81)	10 (18)	287 (92)	< 0.001	ref
Yes 70 (19)	45 (82)	25 (8.0)		52 (23–110)
TTP				
> 12 h 162 (44)	4 (7.3)	158 (51)	< 0.001	ref
**≤** 12 h 205 (56)	51 (93)	154 (49)		13 (4.6–37)

^a^The number of episodes and the share of episodes are given within parenthesis

^b^Univariate test of significance was performed with the chi-square test

^c^Not applicable since calculation of odds ratio was impossible due to perfect separation

A sensitivity analysis performed by removing all recurrent episodes from the analysis and leaving only the first episode in each patient (*n* = 323), showed similar results, OR for IE was 13.4 (95% CI 4.0 to 45.2) adjusted for age, gender, Charlson score, and nosocomial acquisition (see supplementary table 2 for univariate analyses).

### Outcomes

The overall 30-day mortality was 11% (41 patients) and the TTP was similar between survivors and non-survivors (Table 4). In-hospital mortality was 14% and 180-day mortality was 24% and TTP was similar between the groups also using these alternative outcome-measures (data not shown).

**Table 4:** TTP and outcomes

Outcome	TTP median (IQR)	*p* value
30 day mortality (*n* = 41, 11%)	11.0 (9.8–12.7)	0.35
30 day survival (*n* = 326, 89%)	11.7 (9.9–14.1)	
Relapse of bacteremia (*n* = 44, 12%)	10.8 (9.0–13.5)	0.26
No relapse of bacteremia (*n* = 323 s, 88%)	11.7 (10.0–14.1)	

A new EFsB was detected after 44 episodes. The median TTP in episodes followed by recurrence was 10.8 h (IQR 9.0–13.5), compared to 11.7 (IQR 10.0–14.1) in episodes without later recurrence. This difference was not statistically significant. Recurrences occurred in 32 patients who experienced two episodes (*n* = 23), three episodes (*n* = 6), or four episodes (*n* = 3), respectively. In ten patients who did not undergo TEE during the first episode, IE was recognized in a following episode of EFsB.

## Discussion

In this retrospective study, we found that a short TTP in EFsB is associated with a higher risk of IE, whereas TTP was not associated with prognosis. TTP is a readily available parameter which potentially contains important information about the biomass of bacteria in blood. Since IE is an intravascular infection, it appears reasonable that the number of bacteria in blood is higher, and thus TTP shorter, than in patients with other foci of infection with simultaneous bacteremia. A low TTP has previously been associated with IE in *S*. *aureus* bacteremia and here we demonstrate that TTP is also significantly lower in *E*. *faecalis* IE than in other types of *E*. *faecalis* bacteremic infections. Importantly, IE was the only factor associated with a low TTP. A growing body of evidence suggests that IE is common in EFsB and therefore, transesophageal echocardiography is often needed in cases with a risk for IE. The DENOVA score has previously been described as sensitive and specific for IE, but TTP might provide additional information to the decision on whether to perform transesophageal echocardiography. We found that TTP alone is a predictor of IE with a high sensitivity (93%), but a relatively low specificity (51%) using a cut-off at ≤ 12 h. Thus, already when conveying the message of a positive blood culture, the laboratory could potentially signal the risk for IE in patients with EFsB based on the number of positive cultures, the TTP, and whether the culture is mono- or polymicrobial. If the TTP is longer than 12 h, IE is likely a rare event and this information is important since further work-up with transesophageal echocardiography might not be needed in most cases.

The mortality in our cohort was lower than previously reported e.g. from Denmark [13]. We believe that this at least in part is due to a lower proportion of nosocomial infections, which typically have a higher mortality rate, in our cohort. As opposed to previous studies on bacteremia caused by several different pathogens, we did not find an association between TTP and prognosis. Since our study is relatively large, we conclude that TTP is not a clinically important feature in determining the risk for death in EFsB. The association between TTP and IE, and lack of association between TTP and mortality is likely explained by the fact that mortality in *E*. *faecalis* IE was similar to that of EFsB not complicated by IE in our cohort. However, it is still very important to identify and treat IE properly, but if this is done correctly, the prognosis might not be worse than for EFsB with other infection foci.

The results from this study should be interpreted in the light of its retrospective design which limits the information about episodes to what has been noted in medical records. Importantly, since not all patients underwent TEE, there is a risk that IE could have been missed. The fact that ten recurrent episodes were diagnosed with IE indicates that this indeed happened in our cohort. Such potential misclassification of patients with IE as non-IE could lead both to a risk of overestimation of sensitivity and underestimation of specificity. It is important to keep this problem in mind when applying the results in practice and not solely rely on TTP or any given clinical variable in the complex process of deciding upon optimal clinical management.

The TTP in itself is highly sensitive to several different, potentially systematic, variations. For example, previous administration of antibiotics might increase TTP and we chose to exclude those patients. Therefore, our results cannot be extrapolated to patients that already received antibiotics. In order to decrease variations in the time from the sampling of blood to the start of incubation of the blood culture vials, we only included cultures from the hospitals that had procedures to ensure that blood cultures are put into a cabinet at all hours. Moreover, the blood volume in the vials might also influence TTP. We believe that these variables are mainly non-differential and they could have been controlled in a prospective study. Despite all these variations, TTP was still found to be associated to IE. This demonstrates that the use of TTP in clinical decision-making is feasible using normal clinical routines. Great care must be taken when extrapolating the results from this study to other laboratories and health care systems. The absolute TTP will probably vary depending not only on logistics, but also on blood culture systems. Therefore, external validation of our results is needed before they are implemented in clinical practice.

## Supplementary Information

ESM 1 (DOCX 23 kb)
