# Supplementary material for: Short time to blood culture positivity in Enterococcus faecalis infective endocarditis
Source: Eur J Clin Microbiol Infect Dis. 2021 Mar 9;40(8):1657–64. doi: 10.1007/s10096-021-04210-9 (PMC8295074; doi:10.1007/s10096-021-04210-9)
Supplement: Supplementary file 1 — (DOCX 23 kb) [file 10096_2021_4210_MOESM1_ESM.docx]

Supplementary table 1. sensitivity analysis

| Univariate analysis | | | | | Multivariable analysis^c^ |
| --- | --- | --- | --- | --- | --- |
|  | TTP ≤ 12h (n=174) | TTP > 12h (n=149) | p-value^b^ | OR univariate | OR (95% CI) |
| **Gender** |  |  |  |  |  |
| Male (234, 72%) | 123 (71) ^a^ | 111 (75) | 0.45 | ref | ref |
| Female (89, 28%) | 51 (29) | 38 (26) |  | 1.2 (0.74 – 2.0) | 1.5 (0.85 – 2.6) |
| **Age** |  |  |  |  |  |
| 18-70 (99, 31%) | 50 (29) | 49 (33) | 0.15 | ref | ref |
| 71-80 (122, 38%) | 61 (35) | 61 (41) |  | 0.98 (0.58 – 1.7) | 1.0 (0.59 – 1.9) |
| 81-96 (102, 32%) | 63 (36) | 39 (26) |  | 1.6 (0.90 – 2.8) | 1.7 (0.93 – 3.3) |
| **Charlson score** |  |  |  |  |  |
| 0-2 (186, 58%) | 100 (58) | 86 (58) | 0.064 | ref | Ref |
| 3-4 (82, 25%) | 51 (29) | 31 (21) |  | 1.6 (0.90 – 3.0) | 1.6 (0.91 – 2.8) |
| >5 (55, 17%) | 23 (13) | 32 (22) |  | 2.3 (1.1 – 4.6) | 0.76 (0.40 – 1.5) |
| **Site of acquisition** |  |  |  |  |  |
| Community acquired (269, 83%) | 148 (85) | 121 (81) | 0.36 | ref | ref |
| Nosocomial (54, 17%) | 26 (15) | 28 (19) |  | 0.76 (0.42 – 1.4) | 1.1 (0.57 – 2.1) |
| **Site of infection** |  |  |  |  |  |
| Urinary tract (126, 39%) | 63 (36) | 63 (42) | <0.001 | ref | ref |
| IE (39, 12%) | 36 (21) | 3 (2) |  | 12 (3.5 – 41) | 13 (3.7 – 44) |
| GI and biliary (28, 9%) | 12 (7) | 16 (11) |  | 0.75 (0.33 – 1.7) | 0.74 (0.31 – 1.8) |
| Skin and soft tissue (23, 7%) | 11 (6) | 12 (8) |  | 0.92 (0.38 – 2.2) | 0.94 (0.37 – 2.4) |
| Skeletal and joint (13, 4%) | 5 (3) | 8 (5) |  | 0.63 (0.19 – 2.0) | 0.70 (0.21 – 2.3) |
| Other known (10, 3%)^d^ | 4 (2) | 6 (4) |  | 0.67 (0.18 – 2.5) | 0.56 (0.15 – 2.2) |
| Unknown (84, 26%) | 43 (25) | 41 (28) |  | 1.0 (0.60 – 1.8) | 0.98 (0.55 – 1.7) |

Sensitivity analysis only including the first episode of bacteremia per patient. ^a^The number of episodes and the share of episodes are given within parenthesis. ^b^Univariate test of significance was performed with the chi-square test. ^c^The multivariable testing was performed using binary logistic regression. The following variables were included in the model: Gender, age, Charlson score, site of acquisition and site of infection. ^d^pneumonia (n=7), other airway infections (n=3).

Supplementary table 2. sensitivity analysis

|  | IE (n=39) | Non-IE (n=284) | p-value^b^ | OR |
| --- | --- | --- | --- | --- |
| Gender |  |  |  |  |
| Male 234 (72) | 31 (80)^a^ | 203 (72) | 0.29 | ref |
| Female 89 (28) | 8 (21) | 81 (29) |  | 0.65 (0.29 – 1.5) |
| Age |  |  |  |  |
| 18-70 99 (31) | 13 (33) | 86 (30) | 0.93 | ref |
| 71-80 122 (38) | 14 (36) | 108 (38) |  | 0.86 (0.38 – 1.9) |
| 81-96 102 (32) | 12 (31) | 90 (32) |  | 0.88 (0.38 – 2.0) |
| Charlson score |  |  |  |  |
| 0-2 186 (58) | 28 (72) | 158 (56) | 0.069 | ref |
| 3-4 82 (25) | 9 (23) | 73 (26) |  | 0.70 (0.31 – 1.5) |
| >5 55 (17) | 2 (5) | 53 (19) |  | 0.21 (0.049 – 0.92) |
| Site of acquisition |  |  |  |  |
| Community 269 (83) | 37 (95) | 232 (82) | 0.039 | ref |
| Nosocomial 54 (17) | 2 (5) | 52 (18) |  | 0.24 (0.056 – 1.0) |
| Duration of symptoms >7 days |  |  |  |  |
| No 281 (87) | 6 (15) | 275 (97) | <0.001 | ref |
| Yes 42 (13) | 33 (85) | 9 (3) |  | 168 (56 – 502) |
| Embolisation |  |  |  |  |
| No 311 (96) | 27 (69) | 284 (100) | <0.001 | NA^c^ |
| Yes 12 (4) | 12 (31) | 0 (0) |  | NA |
| Number of positive blood cultures >2 |  |  |  |  |
| No 119 (37) | 3 (8) | 116 (41) | <0.001 | ref |
| Yes 204 (63) | 36 (92) | 168 (59) |  | 8.3 (2.5 – 28) |
| Origin of infection |  |  |  |  |
| Known 177 (55) | 1 (3) | 176 (62) | <0.001 | ref |
| Unknown 146 (45) | 38 (97) | 108 (38) |  | 62 (8.4 – 458) |
| Valve disease |  |  |  |  |
| No 261 (81) | 13 (33) | 248 (87) | <0.001 | ref |
| Yes 62 (19) | 26 (67) | 36 (13) |  | 14 (6.5 – 29) |
| Auscultation of murmur |  |  |  |  |
| No 270 (84) | 7 (18) | 263 (93) | <0.001 | ref |
| Yes 53 (16) | 32 (82) | 21 (7) |  | 57 (23 – 145) |
| TTP |  |  |  |  |
| > 12 h 149 (46) | 3 (8) | 146 (51) | <0.001 | ref |
| ≤ 12 h 174 (54) | 36 (92) | 138 (49) |  | 13 (3.8 – 42) |

Sensitivity analysis only including the first episode of bacteremia per patient. ^a^The number of episodes and the share of episodes are given within parenthesis. ^b^Univariate test of significance was performed with the chi-square test. ^c^Not applicable since calculation of odds ratio was impossible due to perfect separation.
